# Family is all that matters: Prospective associations between structure, function, and quality of social relations and self-rated health in the National Social Life, Health, and Aging Project (NSHAP)

**DOI:** 10.1016/j.ssmph.2024.101715

**Published:** 2024-10-10

**Authors:** T.C. Abreu, J.D. Mackenbach, J.W.J. Beulens, I. Vaartjes, I. Kawachi

**Affiliations:** a Department of Epidemiology & Data Science, Amsterdam UMC - Location VUmc, Amsterdam, Noord-Holland, the Netherlands; b Upstream Team, the Netherlands; c Amsterdam Public Health, Amsterdam Cardiovascular Sciences, Amsterdam, Noord-Holland, the Netherlands; d Julius Center for Health Sciences and Primary Care, University Medical Center Utrecht, Utrecht, the Netherlands; e Department of Social and Behavioral Sciences, Harvard School of Public Health, Boston, USA

**Keywords:** Social interactions, Social relationships, Social isolation, Social ties, Social strain, Mental health, General health, Subjective health

## Abstract

We prospectively examined the association between structure, function, and quality of social relations and self-rated health (SRH) in U.S. adults followed over 10 years in the population-based National Social Life, Health, and Aging Project (NSHAP). Large social network and high positive/negative social support were measured at baseline and defined as the highest quartile. These three binary measures were reported from friends, family, and partner and combined into a multifactorial exposure variable. SRH was measured through a 5-point Likert scale and dichotomised. Odds ratios (OR) for poor SRH were estimated with covariate-adjusted logistic regression. In total, 1,592 participants were included. Based on the combined multifactorial exposure variable as well as independent exposure variables, only lower levels of negative social support were prospectively associated with better SRH (aOR = 0.65; 95%CI 0.44–0.98). From the different social ties, only family-related negative social support was associated with poor SRH (aOR = 0.59; 95%CI 0.39–0.90). This association was similar between genders, but only statistically significant among women. Sensitivity analysis with depressive symptoms as outcome supported the hypothesis that the findings for SRH may be partially driven by mental health (aOR = 0.65; 95%CI 0.48–0.90). Concluding, negative social support particularly from family is prospectively associated with poor SRH.

## Highlights

• Prospective study on social relations based on a combined multifactorial exposure variable.
• Lower levels of negative social support associated with reduced likelihood of poor SRH.
• A specific source of negative social support (i.e., family) was associated with poor SRH.
• Social network size and positive support less strongly associated with SRH.
• Only nuanced gender differences, with a stronger association among women.

## 1 Introduction

An extensive evidence base supports the hypothesis that social relations influence a broad range of health outcomes (Holt-Lunstad, 2018; House et al., 1988; Tay et al., 2013), including quality of life, morbidity, and mortality (Gouveia et al., 2016; Holt-Lunstad et al., 2010; Kuiper et al., 2015). In particular, studies have shown robust associations between social connectedness and mental health outcomes (e.g., depression (Kawachi et al., 2001; Santini et al., 2015)). Theoretical explanations for the association between social relations and health include the provision of social support, social influence, and access to resources and material goods through social relations (e.g., facilitating access to better healthcare) (Berkman et al., 2000). These factors influence health through more downstream pathways, which can include indirect behavioral and psychological mechanisms, or direct physiological mechanisms (Berkman et al., 2000; Umberson et al., 2010). For instance, social relations can support or discourage certain lifestyle behaviours (e.g., one's social network discourages smoking behaviour), be a source of self-esteem, or directly influence physiological stress responses (e.g., buffer harmful physiological responses to stressors) (Berkman et al., 2000; Uchino, 2006; Umberson et al., 2010).

A variety of definitions and measurements of social relations are employed in the literature across different disciplines. A variety of definitions and measurements of social relations are employed in the literature across different disciplines. Common across definitions is the recognition of three distinct sub-components of social relations, namely structure, function, and quality (Due et al., 1999; Holt-Lunstad, 2022). Epidemiological studies typically focus on one or two of these dimensions (e.g., social network size and exchange of social support across social ties), but seldom attempt to provide a more comprehensive picture. For example, studies generally find a “dose-response” relation between network size and health outcomes, such as risk of future mortality. However, the size of networks could also be a health liability (role overload) for some individuals, and hence it is necessary to take into account not only network size but also the quality of social support (positive or negative) that is exchanged across different social connections. Our study represents an attempt to integrate the dimensions of network size, function and quality to more fully understand the influence of social ties on health. With regard to structural aspects, social network characteristics such as size and composition have been linked to health outcomes (Cohen, 2004; Lee et al., 2023; Schutter et al., 2022a). In general, smaller social networks are associated with adverse health outcomes (Cohen, 2004), with a recent meta-analysis showing that smaller social networks are related to a higher mortality risk (Schutter et al., 2022a). It is hypothesized that larger social networks can enhance the possibilities of accessing social support resources, although, above a certain threshold, additional social ties may not provide health benefits (Chin et al., 2020). Another structural component, the composition of social networks can encompass different relationship sources (e.g., friends, family, partner or acquaintance), with closer ties being key providers of social support and assumed to be the ties most influential to health (Lee et al., 2023). With regard to functional aspects, the impact of social support from one's social network on health outcomes has been examined for decades (Broadhead et al., 1983). Overall, the literature shows that individuals reporting lower levels of social support present worse health outcomes (Bedaso et al., 2021; Campos, 2015; Cohen, 1988; Holt-Lunstad et al., 2010; Mohd et al., 2019; Nausheen et al., 2009; Smith et al., 2017). For instance, social support is linked to several health outcomes, from lifestyle behaviours (Smith et al., 2017) to disease progression (Nausheen et al., 2009) and mortality (Holt-Lunstad et al., 2010), and in different populational groups, contexts and geographies (Bedaso et al., 2021; Campos, 2015; Mohd et al., 2019).

While the literature has mostly focused on structural and functional components of social relations, more recently qualitative aspects of social relations (e.g.*,* positive *and* negative aspects of social support) are receiving attention. Positive social support is believed to buffer the deleterious health effects of exposure to stress (Cobb, 1976). By contrast, social support may also present itself as negative, which is referred to in the literature by several terms such as social hindrance, social restraints, social strain, problematic support, or negative social exchanges. Yet, few studies have attempted to conceptualise negative social support. Brooks and Schetter (2011) refer to it as social negativity (Brooks et al., 2011), which is defined as the type of support that is perceived as either aversive or unwanted. Dimensions of negative social support include interference (e.g., behaviours that hinder one's capacity to pursue goals) and conflict (e.g., behaviours that elicit anger) (Brooks et al., 2011), which have been associated with deleterious health outcomes, including increased risk of mortality (Lund et al., 2014). However, despite the recent increase in research (Brooks et al., 2011; Offer, 2021), existing research addressing social relations quality has largely focused on positive aspects, while negative aspects remain underexplored, particularly in longitudinal studies (Hakulinen et al., 2016; Offer, 2021). Overall, those studies that do differentiate between positive and negative relationships aspects suggest a differential effect on health, with the effects of negative aspects surpassing or at least being comparable to those of positive aspects on health (Rook, 2015). It is noteworthy that individuals can be simultaneously exposed to both positive and negative social support (e.g., from different relationship sources or in ambivalent/conflictual relationships), and one unresolved question is whether positive or negative social relation quality matters more for health.

Although these three components have been individually investigated in relation to health outcomes, studies have only recently begun to unpack their joint contribution to health. As dynamic constructs, these three components may overlap and display compensatory or trade-off mechanisms (Doyle et al., 2024); however, it is still unclear whether structural, functional, or qualitative aspects of social relations, or a combination of them, matter most for health (Holt-Lunstad et al., 2017; Kauppi et al., 2018). For example, few studies have examined the joint effects of social network size (i.e., social relations structure) and quality of social support (i.e., social relations quality) (Fiorillo et al., 2011); whether possessing an extensive social network with high levels of positive social support from some social ties could mitigate the adverse health consequences of exposure to negative social support from other social ties.

Moreover, other important research gaps remain. First, the relationship source of negative social support might also matter for health. While a potential differential impact of social support from different relationship sources on health remains largely understudied, there is evidence to suggest that some relationships can be more impactful than others (Woods et al., 2020). For instance, a 20-year longitudinal study compared the impact of different support measures from different relationship sources on health outcomes and observed an effect of non-marital family support, whereas no effect from partner support was observed (Woods et al., 2020). Second, studies have not fully explored potential heterogeneity in these associations across population sub-groups, particularly by gender. Although there are widely known gender differences in health outcomes (Schutter et al., 2022b) and gender-patterned social roles in society (Gabriel et al., 1999; Sharma et al., 2016), potential gender-based differences in the role of social relations on health outcomes are still understudied.

In the present study, we sought to prospectively examine the association between structural, functional, and qualitative aspects of social relations of social relations and self-rated health in a sample of older U.S. adults.

## 2 Methods

### 2.1 Data and subjects

We used data from the National Social Life, Health, and Aging Project (NSHAP) (O'Muircheartaigh et al., 2009; O'Muircheartaigh et al., 2021). NSHAP is a nationally representative population-based study that focuses on health and social relations, aiming at older, community-dwelling individuals from the United States. Data was collected across three waves of data collection spanning 10 years, starting in 2005–2006. The protocol was approved by the Institutional Review Boards of the University of Chicago and the National Opinion Research Center and was in accordance with the Declaration of Helsinki. All respondents provided written informed consent.

**Fig. 1:**
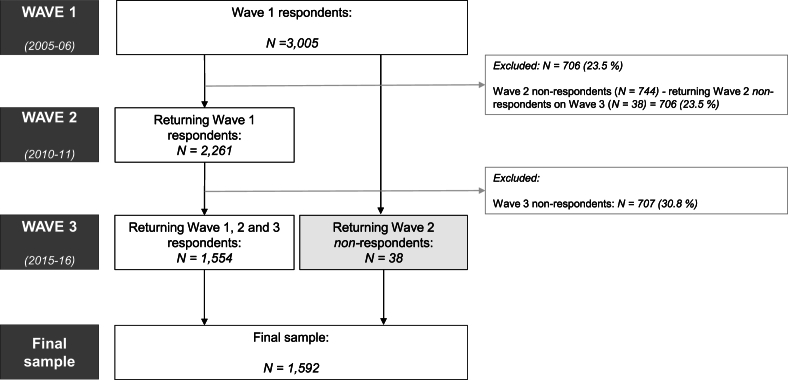
Flow diagram depicting the study inclusion criteria.

The sample was selected using a multi-stage area probability design, with oversampling according to race/ethnicity, age, and gender. The sample at baseline includes 3005 participants (75.5% response rate), aged 57–85 years (O'Muircheartaigh et al., 2021). Only participants who responded both to wave 1 (2005–06) and wave 3 (2015–16) were included in the present analyses (*N* = 1592), which excluded 1413 respondents (47% sample attrition) (Fig. 1). Partners of respondents were not included because they entered the study from wave 2 onward.

### 2.2 Measures

#### 2.2.1 Exposure variables

We used a series of variables to measure social network size and social support from three major sources of social relationships: close family, friends, and partner. The exposure variables were constructed using data collected at wave 1. In this paper, we adopt the term “exposure”, which is used interchangeably with “independent variable” within the context of a regression framework.

To quantify the social network size of each participant, we used self-reported responses to three questions: (1) whether the respondent has a spouse or partner (*i.e.,* romantic, intimate, or sexual partner), herein referred to as partner, (2) the number of friends, and (3) the number of close family members (*i.e.,* a family member or relative to whom the respondent feels close, excluding their partner). For friends and family, respondents could choose one of the following six response categories: none, 1, 2–3, 4–9, 10–20, or more than 20. Due to the non-continuity nature of these response categories and the need to ensure comparability across the responses for friends and family, the answers to these questions were standardized (i.e., transformed into z-scores) and then scores from family and friends were summed and averaged. Acknowledging that having a partner contributes to a larger social network, participants reporting having a partner at baseline were assigned an additional point. Participants with small networks were underrepresented in our sample, hence we combined those with small to moderate size and compared them against participants with the largest social networks. We dichotomized the final score, such as scores equal to or above the third quartile were categorized as large social network, while those below were considered small to moderate.

**Fig. 2:**
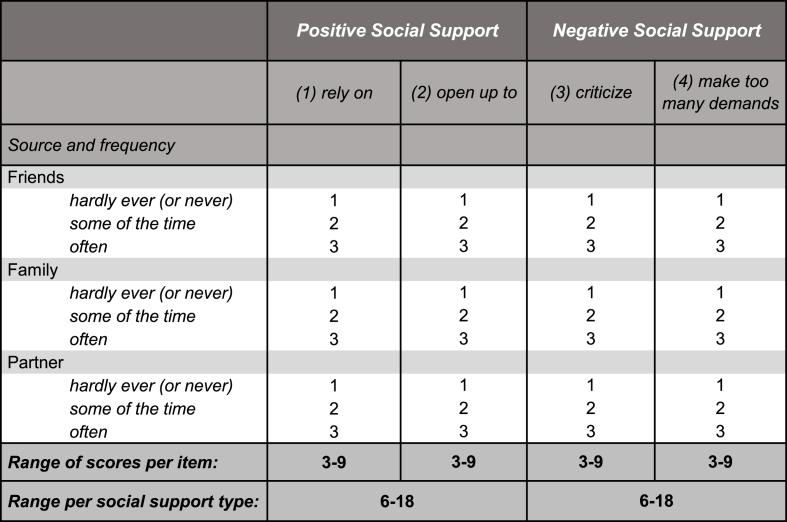
Representation of scores assigned to frequency of social support per relationship type, for positive and negative social support.

Positive social support and negative social support were assessed separately for three different intimate relationships: close family members, friends and partner. Positive social support was assessed by two items: how often could respondents (1) open up to, and (2) rely on their family members/friends/partner. Negative social support was assessed by the two items: how often family members/friends/partner (3) criticize them, and (4) make too many demands on them. All items could be rated by the participants on a 3-point scale indicating frequency, which was assigned the value of 1 to “never or hardly ever”, 2 to “some of the time”, and 3 to “often” (Fig. 2). In order to obtain a total score, the relationship-specific scores were summed separately for positive and negative social support scales (total score range from 6 to 18). In case a participant reported not having a partner, a score of 1 (corresponding to receiving support “never or hardly ever”) was added to the items referring to partner support. The positive and negative social support scores were then dichotomized according to quartiles, *i.e.,* scores equal to or above the third quartile were categorized as high support (hypothesized to be the category most influential to health), while those below are considered low to moderate support.

**Fig. 3:**
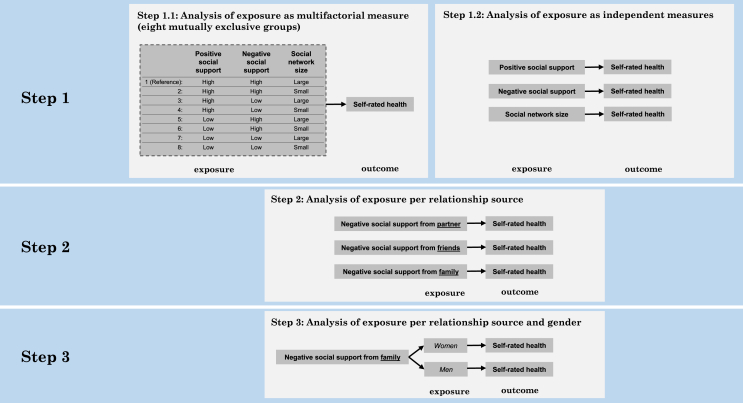
Stepwise analytical approach illustrating the refinement of exposure measures.

To comprehensively assess the combined associations of structural, functional and qualitative measures of social relations, we constructed a multifactorial independent variable that encompasses the three social relations measures described above. Because investigating a combination of different exposure measures would be challenging in a continuous fashion, we combined the dichotomous measures of social network size (small vs. large), positive social support (low vs. high) and negative social support (low vs. high) into a single variable with eight mutually exclusive categories, each representing a unique combination of the three original measures. Each participant was assigned to one out of eight possible combinations, for instance, “high positive social support - high negative social support - large social network” (see **step 1.1** in Fig. 3).

#### 2.2.2 Dependent variable

The primary outcome variable was self-rated health (SRH) at wave 3. SRH is widely used in the literature to explore respondents’ health because it is a subjective measure that it is a powerful predictor of health-related outcomes such as mortality (Benyamini, 2011; Idler et al., 1997). The respondents rated their self-assessed general physical health on a five-point scale (ranging from “poor” to “excellent”), with higher scores indicating higher self-rated physical health. Because the lack of linearity across the response options did not allow self-rated health to be analysed in a continuous form, it is commonly dichotomized in the literature (Cullati et al., 2020). We dichotomized SRH into “*good”* (comprising “excellent”, “very good”, “good”) and “*poor”* (comprising “fair” and “poor”), with *poor* SRH as the outcome of interest.

#### 2.2.3 Covariates

Covariates included are age (continuous variable), gender (female vs. male), partner status (single vs. married/partnered), education (less than high school, high school or some degree), race/ethnicity (White vs. Black, Hispanic, or other), subjective household income relative to American families (below average income, average income or above average income), employment status (working vs. not working), and SHR measured at baseline.

We modelled the variable “partner status” both as part of the exposure variable and as a control variable due to its potential to independently influence self-rated health beyond the effects captured by our exposure variables. In the preliminary analysis, we found that excluding “partner status" from the construction of the exposure variables had minimal impact on the group assignment of participants. Similarly, removing “partner status" from the set of control variables resulted in only a marginal change in exposure estimates.

An additional covariate was the frequency of depressive symptoms, which was measured with the Center for Epidemiological Studies Depression Scale (CES-D) (Kohout et al., 1993). CES-D was assessed by an 11-item short form, where answers were rated on a 3-point frequency scale ranging from “rarely or none of the time” to “most of the time” (Payne et al., 2014). Scores were summed and then dichotomized, with scores ≥9 being categorized as frequent depressive symptoms. All covariates were self-reported and measured at baseline, except CES-D which was measured at wave 3.

#### 2.2.4 Statistical analysis

All results were presented based on weighted data. Weights from wave 1 accounted for the complex survey design (*i.e.,* sample clustering and stratification), for selection probabilities, and non-response (O'Muircheartaigh et al., 2009).

Based on descriptive statistics, some variables presented considerable missingness, notably with 14% of participants lacking data on at least one of the exposure variables and 18% on subjective income. Therefore, we performed multiple imputation to handle missingness. With Multivariate Imputation by Chained Equations (R package *mice*), we created 10 imputed datasets replacing missing values with imputed data. Following imputation, analyses combined observed and imputed data and included all participants.

Descriptive statistics were presented for all measures, using mean and standard deviation (SD) for continuous variables and frequency rates (%) for categorical variables, both with imputed weighted data.

Logistic regression models were used to investigate the association between the quality and quantity of social relations and poor SRH in a stepwise approach starting with the multifactorial exposure variable and exploring which aspects of social relations, coming from which social ties, were most relevant for SRH, based on statistical significance (Fig. 3).

To assess the combined associations of structural, functional and qualitative measures of social relations, for **step 1.1**, we modelled the multifactorial aspect of social relations using the eight mutually exclusive exposure categories described above. In **step 1.2**, we assessed each exposure variable independently (i.e., we modelled each of the three social relation variables in a separate model). Informed by the insights gained from **steps 1.1** and **1.2** (see Results section for details), in **step 2** we solely focused on the exposure variable negative social support**.** We further examined negative social support by exploring the differential effect of sources of support on SRH. Hence, we stratified negative social support according to relationship source, namely family, friends and partner. Because previous research has shown gender differences in the association between social relations and health (Caetano et al., 2013; Glynn et al., 1999), we examined potential gender differences in our study. Based on the findings of the stepwise approach described above, in **step 3** we tested an interaction term between gender and the exposure variable negative social support from the family as well as stratified the final model by gender. In all steps, we established the exposure category hypothesized to be “worst-off” as the reference category *(*e.g.*, low/moderate* positive social support*, high* negative social support*, and small/moderate* social network*)*.

Sensitivity analyses were conducted with a mental health outcome: depressive symptoms. We assessed the association between the exposure variable from **step 3** in Fig. 3 (*i.e.,* negative social support from the family) and frequent depressive symptoms (*i.e.,* CES-D ≥ 9). This sensitivity analysis tested the hypothesis that SRH scores may be partially driven by mental health. Finally, to assess the robustness of our findings, we conducted sensitivity analysis substituting the original dichotomized exposure measures with alternative exposure variables in a continuous fashion.

Odds ratios (OR) with 95% confidence intervals (CI) were obtained for crude and adjusted models. The models were adjusted incrementally, first for age and gender (Model 1), then for partner status, education, race/ethnicity, income, and employment status (Model 2), and finally for self-rated health at baseline (Model 3). The final models were further adjusted for depressive symptoms (Models 4–7). All statistical analyses were conducted using R version 4.2.1, and statistical significance was considered at α = 0.05.

## 3 Results

Key demographic characteristics of respondents were summarized in Table 1 (weighted and imputed data). In the present study, 1592 participants were included. At baseline, the average age was 65.7 ± 6.4 years and women constituted 54% of respondents. The majority of participants were White (81%), pursued education beyond high school (61%), and were married or had a partner (78%). At wave 3, 22% reported poor SRH and 23% reported frequent depressive symptoms. The distribution of participants across the eight distinct categories of the exposure variable ranged from 1% to 56% (Table 1).

**Table 1:** Sociodemographic characteristics for the analytical sample, NSHAP (imputed and weighted sample, n = 1592)^a^^,^^b^.

Characteristics	Mean or N	±SD or %
**Age** (years)	65.7	6.4

**Gender**		
men	730	46%
women	862	54%

**Educational level**		
less than high school	227	14%
high school diploma	397	25%
some degree or more	968	61%

**Ethnic group**		
Black, Hispanic, or other race	302	19%
White	1290	81%

**Partnered (married or partnered)**		
no	351	22%
yes	1241	78%

**Employment status**		
not employed	681	43%
employed	911	57%

**Subjective income**		
(far) below average	444	28%
average	639	40%
(far) above average	509	32%

**Self-rated health**		
*Wave 1*		
good	1314	83%
poor	278	17%
*Wave 3*		
good	1234	78%
poor	358	22%

**Depressive symptoms:** mean score	5.8	4.3

**Frequent depressive symptoms**		
frequent	363	23%
no or infrequent symptoms	1229	77%

**Positive social support:** mean score	14.2	2.5

**Positive social support**		
low/moderate	1257	79%
high	335	21%

**Negative social support:** mean score	7.7	1.7

**Negative social support**		
low/moderate	1338	84%
high	254	16%

**Network size**		
small/moderate	1211	76%
large	381	24%
**Mutually exclusive groups of exposure**^c^		
High Positive, High Negative, Large Network	14	1%
High Positive, High Negative, Small Network	26	2%
High Positive, Low Negative, Large Network	103	6%
High Positive, Low Negative, Small Network	136	9%
Low Positive, High Negative, Large Network	37	2%
Low Positive, High Negative, Small Network	169	11%
Low Positive, Low Negative, Large Network	214	13%
Low Positive, Low Negative, Small Network	893	56%

a Estimates are weighted using NSHAP person weights adjusted for complex survey design (i.e., sample clustering and stratification), selection probabilities, and non-response.

b All variables refer to wave 1, except for self-rated health (wave 1 and wave 3) and depressive symptoms (wave 3 only).

c Positive = Positive Social Support; negative = Negative Social Support; Network = Social Network Size.

In Table 2, we assessed the odds ratios of poor SRH for the multidimensional exposure variable. Overall, only the categories including individuals with “low negative social support” were significantly associated with poor SRH compared with the reference category (*i.e., “low* positive social support *- high* negative social support *- small* social network*”*), irrespective of the level of positive social support or the social network size. This implies that, in combination with other qualitative and quantitative measures of social relations, negative social support is the primary determinant of poor SRH. This pattern is consistently observed across all models, except for the category “*low* positive social support - *low* negative social support - *large* social network” in the fully adjusted model (Model 3: OR = 0.64; 95% CI 0.32–1.27). Moreover, as indicated by ORs of similar magnitude across categories with the same levels of positive social support and negative social support but varying levels of social network size, social network size does not seem to largely influence SRH (e.g.*, “low* positive social support *- low* negative social support *- large* social network*”* OR = 1.88 *vs. “low* positive social support *- low* negative social support *- small* social network*”* OR = 1.95). We caution that the category *“high* positive social support *- high* negative social support *- large* social network*”* contains only 14 participants, therefore estimates for this category are imprecise (Table 2).

**Table 2:** ORs for the association between eight mutually exclusive categories and self-rated health (ORs, 95%CI)^a^.

	Model 1		Model 2		Model 3	
	OR	95%CI	OR	95%CI	OR	95%CI
(Intercept)	0.08	(0.03–0.26)	1.98	(0.34–11.6)	0.15	(0.02–1.24)
Mutually exclusive categories of exposure^b^ (ref = Low Positive, High Negative, Small Network)	1.00	–	1.00	–	1.00	–
High Positive, High Negative, Large Network	**0.04**	**(0.01**–**0.22)**	**0.04**	**(0.01**–**0.25)**	**0.07**	**(0.01**–**0.42)**
High Positive, High Negative, Small Network	0.33	(0.09–1.16)	0.38	(0.10–1.43)	0.53	(0.13–2.16)
High Positive, Low Negative, Large Network	**0.28**	**(0.15**–**0.52)**	**0.27**	**(0.14**–**0.53)**	**0.38**	**(0.18**–**0.82)**
High Positive, Low Negative, Small Network	**0.31**	**(0.18**–**0.53)**	**0.35**	**(0.19**–**0.63)**	**0.46**	**(0.23**–**0.95)**
Low Positive, High Negative, Large Network	0.71	(0.26–1.91)	0.70	(0.27–1.80)	0.71	(0.31–1.65)
Low Positive, Low Negative, Large Network	**0.53**	**(0.33**–**0.86)**	**0.51**	**(0.30**–**0.87)**	0.64	(0.32–1.27)
Low Positive, Low Negative, Small Network	**0.51**	**(0.36**–**0.73)**	**0.46**	**(0.31**–**0.70)**	**0.53**	**(0.33**–**0.86)**
Age (years)	1.03	(1.01–1.05)	1.01	(0.98–1.03)	1.02	(1.00–1.05)
Gender (ref = male)	0.83	(0.60–1.14)	0.68	(0.49–0.94)	0.69	(0.49–0.96)
Female						
Partnered (ref = no partner)			0.93	(0.64–1.36)	1.10	(0.71–1.69)
Partnered						
Education (ref = less than high school)			0.46	(0.32–0.64)	0.62	(0.40–0.96)
High school						
Some degree or more			0.31	(0.20–0.48)	0.48	(0.29–0.78)
Race/Ethnicity (ref = Black, Hispanic, other)			1.17	(0.85–1.62)	1.30	(0.91–1.86)
White						
Subjective income (ref = below average)			0.65	(0.51–0.85)	0.82	(0.62–1.09)
Average						
Above average			0.44	(0.29–0.66)	0.53	(0.34–0.83)
Employment status (ref = unemployed)			0.62	(0.43–0.88)	0.80	(0.54–1.17)
Employed						
Self-rated health at baseline (ref = good)					6.96	(4.43–10.9)
Poor						

a Estimates are weighted using NSHAP person weights adjusted for complex survey design (i.e., sample clustering and stratification), selection probabilities, and non-response.

b Positive = Positive Social Support; negative = Negative Social Support; Network = Social Network Size.

Next, we assessed the relationship of each exposure measure with SRH independently. In fully adjusted models (Models 3), both positive social support and social network size were not independently associated with SRH (OR = 0.67; 95% CI 0.41–1.09 and OR 0.92; 95% CI 0.62–1.38 in Supplementary Table 1 and Supplementary Table 2, respectively). Regarding negative social support, we confirmed its role in influencing SRH by assessing its sole relationship with SRH. We found that lower levels of negative social support were linked to better SRH, i.e., participants who reported higher negative social support had a reduced likelihood of better SRH (Model 3: OR = 0.65; 95% CI 0.44–0.98; Table 3).

**Table 3:** ORs for the association between negative social support and self-rated health (ORs, 95%CI)^a^.

	Model 1		Model 2		Model 3	
	OR	95%CI	OR	95%CI	OR	95%CI
(Intercept)	0.05	(0.02–0.14)	1.50	(0.26–8.65)	0.11	(0.01–0.87)
Negative Social Support (ref = High)	1.00	–	1.00	–	1.00	–
Low	**0.62**	**(0.44**–**0.88)**	**0.57**	**(0.40**–**0.81)**	**0.65**	**(0.44**–**0.98)**
Age (years)	1.04	(1.02–1.05)	1.01	(0.99–1.03)	1.03	(1.00–1.05)
Gender (ref = male)	0.80	(0.58–1.10)	0.64	(0.45–0.90)	0.67	(0.47–0.95)
Female						
Partnered (ref = no partner)			0.84	(0.59–1.19)	1.06	(0.72–1.55)
Partnered						
Education (ref = less than high school)			0.44	(0.31–0.63)	0.61	(0.39–0.94)
High school						
Some degree or more			0.32	(0.21–0.49)	0.48	(0.30–0.78)
Race/Ethnicity (ref = Black, Hispanic, other)			1.15	(0.84–1.59)	1.29	(0.91–1.84)
White						
Subjective income (ref = below average)			0.64	(0.50–0.83)	0.81	(0.61–1.08)
Average						
Above average			0.42	(0.28–0.64)	0.52	(0.33–0.81)
Employment status (ref = unemployed)			0.62	(0.43–0.88)	0.80	(0.55–1.17)
Employed						
Self-rated health at baseline (ref = good)					7.24	(4.61–11.4)
Poor						

a Estimates are weighted using NSHAP person weights adjusted for complex survey design (i.e., sample clustering and stratification), selection probabilities, and non-response.

Subsequently, informed by the findings above that negative social support is the primary determinant of poor SRH, Table 4a, Table 4b, Table 4ca-4c summarizes the regression results according to the source of negative social support (*i.e.,* family, friends and partner). We observed that negative social support from family members is the only source associated with poor SRH (Model 3: OR = 0.59; 95% CI 0.39–0.90), while negative social support from friends and partner were not associated with SRH.

**Table 4a:** ORs for the association between negative social support from family and self-rated health (ORs, 95%CI)^a^.

	FAMILY					
	Model 1		Model 2		Model 3	
	OR	95%CI	OR	95%CI	OR	95%CI
(Intercept)	0.05	(0.02–0.17)	1.41	(0.24–8.21)	0.11	(0.01–0.83)
Negative Social Support (ref = High)	1.00	–	1.00	–	1.00	–
Low	**0.54**	**(0.37**–**0.79)**	**0.54**	**(0.36**–**0.79)**	**0.59**	**(0.39**–**0.90)**
Age (years)	1.04	(1.02–1.05)	1.01	(0.99–1.03)	1.03	(1.00–1.05)
Gender (ref = male)	0.74	(0.53–1.02)	0.60	(0.42–0.86)	0.63	(0.44–0.91)
Female						
Partnered (ref = no partner)			0.92	(0.66–1.30)	1.15	(0.81–1.65)
Partnered						
Education (ref = less than high school)			0.43	(0.30–0.61)	0.60	(0.39–0.92)
High school						
Some degree or more			0.32	(0.21–0.48)	0.49	(0.31–0.78)
Race/Ethnicity (ref = Black, Hispanic, other)			1.15	(0.84–1.57)	1.30	(0.93–1.81)
White						
Subjective income (ref = below average)			0.63	(0.48–0.82)	0.77	(0.58–1.03)
Average						
Above average			0.40	(0.27–0.61)	0.48	(0.31–0.75)
Employment status (ref = unemployed)			0.62	(0.44–0.88)	0.81	(0.56–1.17)
Employed						
Self-rated health at baseline (ref = good)					7.20	(4.58–11.3)
Poor						

a Estimates are weighted using NSHAP person weights adjusted for complex survey design (i.e., sample clustering and stratification), selection probabilities, and non-response.

**Table 4b:** ORs for the association between negative social support from friends and self-rated health (ORs, 95%CI)^a^.

	FRIENDS					
	Model 1		Model 2		Model 3	
	OR	95%CI	OR	95%CI	OR	95%CI
(Intercept)	0.04	(0.01–0.12)	1.07	(0.18–6.34)	0.08	(0.01–0.64)
Negative Social Support (ref = High)	1.00	–	1.00	–	1.00	–
Low	0.87	(0.69–1.11)	0.88	(0.69–1.14)	0.93	(0.74–1.18)
Age (years)	1.03	(1.02–1.05)	1.01	(0.99–1.03)	1.02	(1.00–1.05)
Gender (ref = male)	0.79	(0.57–1.08)	0.64	(0.45–0.90)	0.66	(0.47–0.94)
Female						
Partnered (ref = no partner)			0.89	(0.63–1.25)	1.10	(0.76–1.60)
Partnered						
Education (ref = less than high school)			0.44	(0.31–0.64)	0.62	(0.40–0.96)
High school						
Some degree or more			0.33	(0.21–0.50)	0.50	(0.31–0.81)
Race/Ethnicity (ref = Black, Hispanic, other)			1.10	(0.82–1.49)	1.25	(0.91–1.74)
White						
Subjective income (ref = below average)			0.64	(0.49–0.84)	0.80	(0.60–1.06)
Average						
Above average			0.41	(0.27–0.62)	0.49	(0.32–0.75)
Employment status (ref = unemployed)			0.62	(0.44–0.88)	0.81	(0.56–1.18)
Employed						
Self-rated health at baseline (ref = good)					7.38	(4.73–11.5)
Poor						

a Estimates are weighted using NSHAP person weights adjusted for complex survey design (i.e., sample clustering and stratification), selection probabilities, and non-response.

**Table 4c:** ORs for the association between negative social support from partner and self-rated health (ORs, 95%CI)^a^.

	PARTNER					
	Model 1		Model 2		Model 3	
	OR	95%CI	OR	95%CI	OR	95%CI
(Intercept)	0.04	(0.01–0.11)	1.46	(0.27–7.82)	0.11	(0.02–0.82)
Negative Social Support (ref = High)	1.00	–	1.00	–	1.00	–
Low	0.87	(0.61–1.22)	0.72	(0.48–1.07)	0.75	(0.50–1.12)
Age (years)	1.03	(1.02–1.05)	1.01	(0.98–1.03)	1.02	(1.00–1.05)
Gender (ref = male)	0.80	(0.58–1.10)	0.65	(0.46–0.91)	0.68	(0.48–0.96)
Female						
Partnered (ref = no partner)			0.80	(0.54–1.16)	1.01	(0.67–1.52)
Partnered						
Education (ref = less than high school)			0.43	(0.30–0.62)	0.60	(0.39–0.94)
High school						
Some degree or more			0.32	(0.21–0.50)	0.50	(0.31–0.80)
Race/Ethnicity (ref = Black, Hispanic, other)			1.11	(0.81–1.51)	1.26	(0.90–1.77)
White						
Subjective income (ref = below average)			0.64	(0.50–0.83)	0.79	(0.60–1.04)
Average						
Above average			0.41	(0.27–0.61)	0.49	(0.32–0.74)
Employment status (ref = unemployed)			0.62	(0.44–0.88)	0.81	(0.56–1.17)
Employed						
Self-rated health at baseline (ref = good)					7.33	(4.76–11.3)
Poor						

a Estimates are weighted using NSHAP person weights adjusted for complex survey design (i.e., sample clustering and stratification), selection probabilities, and non-response.

Regarding gender differences, even though the interaction term between negative social support from family and gender was not statistically significant (*p-value* = 0.560), in subsequent gender sub-group analysis we observed that the association between family-negative social support and poor SRH is significant only among women although the effect size is similar among men (Models 3: OR_women_ = 0.56; 95% CI 0.33–0.93 *vs.* OR_men_ = 0.67; 95% CI 0.35–1.29; Supplementary Table 3).

In sensitivity analyses with depressive symptoms, the findings remained largely consistent when compared to poor SRH as the outcome (e.g., Model 3: OR_full sample_ = 0.65; 95% CI 0.48–0.90; Supplementary Table 4). Finally, in further exploring the hypothesis that SRH scores may be partially driven by mental health, we observed that when all models were further adjusted for depressive symptoms, the effect sizes of the exposure measures became non-significant (e.g.*,* Model 6.1: OR = 0.67; 95% CI 0.43–1.04; Supplementary Table 5).

In sensitivity analysis substituting dichotomized exposure measures with exposure variables in a continuous fashion, we observed that the direction of the effect estimates and the pattern in statistical significance remained consistent across the models, while ORs were attenuated (data not shown). Attenuated ORs are expected in this analysis because the ORs represent the association of a one-unit increase in score and are no longer a comparison between low vs. high categories. However, despite largely consistent results for all models, sensitivity analysis showed that the association between family-negative social support and poor SRH became significant among men [continuous exposure: OR 0.77 (95%CI 0.61–0.97) vs. dichotomous exposure presented in Supplementary Table 3: OR 0.67 (95%CI 0.35–1.29)].

In general, crude and adjusted estimates, showed consistent patterns across all models and findings were robust with unweighted and un-imputed data (data not shown).

## 4 Discussion

Our prospective study represents an important addition to the literature on the quality and sources of social relations and health. We highlight four key findings from our study: (1) the size of social networks was less strongly associated with SRH compared to social support, (2) the presence of negative social support was more strongly associated with SRH compared to positive social support, (3) a specific source of negative social support (family versus partner/friends) was most strongly associated with poor SRH, and (4) SRH scores may be partially driven by mental health. 10.13039/100014337Furthermore, in an analytical approach that considered the joint effect of different social relation measures, we found no evidence that high levels of positive social support and large social network size may mitigate the adverse health impacts of exposure to negative social support on SRH. Also, we found only nuanced gender differences, with a stronger association among women.

To our knowledge, this is the first study to assess the joint effect of social network size measures with social support in relation to SRH. With regard to social network size, although the link between a larger social network and decreased mortality risk is well documented (Berkman et al., 1979; Schutter et al., 2022b), other outcomes present mixed findings, for instance, depression (Falci et al., 2009). In relation to social network size and SRH, there are fewer studies (Cornwell et al., 2015; Youm et al., 2014). An earlier study conducted in NSHAP and its “sister” study conducted with Korean subjects (KSHAP) observed that individuals with a larger social network reported better SRH (Youm et al., 2014). In contrast, our findings suggest that varying levels of social network size do not seem to largely influence SRH. These differences might be partially explained by different social network size assessment methods, alternative thresholds, or non-linear relationships such as reported between social network size and wellbeing (English et al., 2014).

In relation to social support, despite the adoption of different definitions of (negative) social support in the literature, which may hamper comparability across studies (Brooks et al., 2011; Gottlieb et al., 2010; Offer, 2021), previous studies have shown an association between negative relationship aspects (or “negativity”, “interpersonal conflicts”, “negative social exchanges”) and SRH (Newsom et al., 2008; Umberson et al., 2006; Wickrama et al., 1997). For instance, similarly to our findings, a prospective study with Dutch participants found that higher negative social support was associated with worse SRH (Croezen et al., 2012). Likewise, Newsom et al. (2022) reported that older adults living in the U.S. having frequent negative “social exchanges” were more likely to experience an accelerated decline in SRH over time (Newsom et al., 2022). Evidence for other health outcomes, such as mental health and quality of life, has also shown negative social support to be associated with adverse health effects (Hakulinen et al., 2016; Strayhorn et al., 2021). Additionally, the stronger association between negative social support and health compared to positive social support is supported by the “negativity bias” theory, where negative social events/exchanges could have a more pronounced effect on health than the benefits produced by positive events (Lazarus, 2021; Rook, 2015). This phenomenon has been observed for other health outcomes (Cox et al., 2016; Geue et al., 2019; Harada et al., 2018).

With regard to different sources of negative social support, a more consequential influence of families on health over other relationship sources (*i.e.,* friends and partner) is plausible given the pivotal lifelong role of family and the persistence of familial relationships. While the dominant influence of family relationships has also been reported for other health outcomes (Shor et al., 2013; Woods et al., 2020), different relationship sources can be more or less influential on health at different points of the life course. Although it is reasonable that individuals would avoid negative ties, that can be particularly challenging for familial ties. While burdensome relationships with a spouse and friends can be transient or dissolved through deliberate decisions (e.g.*,* by divorce), negative ties with children and parents tend to endure due to several factors, including social norms (Offer et al., 2018).

Our study observed nuanced gender differences. While family-negative social support was associated with SRH among men in sensitivity analysis with continuous exposures, the association between social relation measures was more consistent and pronounced among women. Gender differences are expected considering that, besides well-known gender disparities in health (Gottlieb et al., 2010), women experience social life and social expectations distinctively different from men. For instance, women predominantly provide informal care (Nausheen et al., 2009) and are reported to react more strongly to negative stimuli (Newsom et al., 2008) and, in laboratory studies, experience greater physiological arousal than men following arguments between spouses (English et al., 2014). Although several studies on social relations and health have identified these gender differences (Hakulinen et al., 2016; Rook, 2015), the findings, including our own, have been mixed. These inconsistencies highlight the need for further research.

Furthermore, we hypothesized that the SRH measure encompasses not just physiological health aspects, but also mental health aspects, thus the association between social relations and SRH could be partially explained by depressive symptoms. Indeed, depressive symptoms have been extensively documented as being influenced by (negative) social relationships (Kawachi et al., 2001; Santini et al., 2015).

### 4.1 Strength and limitations

We used data from a longitudinal study, which is one of the most comprehensive datasets with information on both quality, quantity and source of social relationships and health. It offers the unique opportunity to elucidate their relationship in a nationally representative sample. However, our study has limitations. Due to our sample size, our study did not allow us to investigate heterogeneity across different racial/ethnic groups. As a common challenge in longitudinal studies, our 15-year follow-up in an older adult population showed important sample attrition between wave 1 and wave 3. Respondents participating in wave 1 but not followed up in wave 3 were more likely to be older, men, not be employed, not have a partner, and have lower income and education. Moreover, they were more likely to experience worse SRH, lower levels of positive social support and smaller social networks. Although weights were employed to account for non-response in wave 1, NSHAP weights accounting for longitudinal non-response were not available at the time of this research and drop-outs between wave 1 and wave 3 potentially yield selection bias.

Moreover, because the sample is restricted to older adults, our findings may not be generalizable to the whole adult population. Furthermore, although the use of longitudinal data helps reduce the concerns about reverse causality, we should be cautious about making causal inferences based on these findings. Considering the potential bidirectionality of effects (*i.e.,* health status could also influence social relations), we adjusted our models to SRH at baseline, which did not substantially change our findings. Although we did not consider longitudinal measures of the exposure variables in our models, the stability of social measures over the 10-year follow-up suggests that this would have a minimal impact on our findings (Cornwell et al., 2021). There could have been also time-varying confounding over the longitudinal follow-up (*i.e.,* an individual's health status may simultaneously mediate the association between social relations measured at time 1 and time 2, as well as confound the association between social relations at time 2 and health status measured at time 3). Additionally, the number of participants included in each of the “mutually exclusive groups of exposure” is unequal and could raise concerns regarding the power and robustness of our findings. However, the conclusions based on the analysis with “mutually exclusive groups of exposure” were supplemented and confirmed by the analysis conducted for each exposure individually, the analysis with continuous exposure measures, and preliminary analysis excluding individuals from the smallest group. Finally, our findings may suffer from social desirability bias, where respondents may have been unwilling to accurately report aspects of their social relations.

### 4.2 Future research

Future studies should continue to differentiate between positive and negative social support and include data with sufficient variability to allow researchers to explore the heterogeneity in social relations according to race and ethnicity (Wong et al., 2023), as well as differences in individualistic and collectivistic cultures (Wu et al., 2021). Such samples may allow for the study of intersectionality (e.g.*,* ethnicity, culture and norms, and gender). Longer follow-up time and larger samples can also allow to explore the dynamics in social relations over time. Moreover, in light of the recent upsurge in digital socialization, it is essential to explore the health impacts of social relations that take place online, which can be particularly relevant for younger age groups. Finally, future research should explore more advanced statistical methods, such as marginal structural models and the g-formula, which can address the potential bi-directional association between exposure and confounders and can estimate the effect of time-varying measures.

### 4.3 Implications

Our findings can inform future research and may offer insights for public policy. Possible public policies aimed at reducing the health consequences of negative social relations may include improving awareness about the importance of patients' social health among healthcare professionals and about the detrimental consequences of negative relationships among the general population. Other possibilities may include improving access to psychological interventions and social prescription (e.g., linking individuals to services and activities that promote health and wellbeing); promoting opportunities to build and strengthen interpersonal relationships (e.g., by fostering social participation); supporting positive social interactions within one's social network, including promoting digital literacy and conscious use of online social networking. Finally, strengthening welfare and housing policies to alleviate the burden of informal care on families, especially on women, and to avoid unwilling multigenerational co-living can be considered.

## 5 Conclusion

Our prospective study provides important insights into how social relationships can impact health. We found that negative social support is the social relation measure most strongly associated with SRH, while positive social support and social network size do not appear to play an important role in SRH among older adults in the U.S. Our findings suggest that negative social support from family is more relevant for SRH than negative social support from other sources. Moreover, the detrimental effect of negative social support on health is partially driven by mental health. Future research on the association between social relations and health is needed, including further examining the role of gender, heterogeneity and intersectionality. We underscore the importance of developing and implementing effective public policies aimed at building and strengthening social relations and promoting measures to mitigate its negative aspects.

## Funding

Financial support for this research was provided by Amsterdam Public Health research institute through a travel grant, EXPOSOME-NL, and EXPANSE. EXPOSOME-NL is funded through the Gravitation program of the Dutch Ministry of Education, Culture, and Science and the Netherlands Organization for Scientific Research (NWO grant number 024.004.017). EXPANSE received funding from the European Union's Horizon 2020 research and innovation program under grant agreement number 874627. The funders had no role in study design, data collection and analysis, decision to publish, or preparation of the manuscript.

## CRediT authorship contribution statement

**T.C. Abreu:** Writing – review & editing, Writing – original draft, Methodology, Formal analysis, Data curation, Conceptualization. **J.D. Mackenbach:** Writing – review & editing, Supervision, Methodology, Conceptualization. **J.W.J. Beulens:** Writing – review & editing, Supervision, Methodology, Conceptualization. **I. Vaartjes:** Writing – review & editing, Supervision, Methodology, Conceptualization. **I. Kawachi:** Writing – review & editing, Supervision, Methodology, Conceptualization.

## Declaration of competing interest

The authors declare that they have no known competing financial interests or personal relationships that could have appeared to influence the work reported in this paper.

## Data Availability

The authors do not have permission to share data.
